# Disseminating Timely Peer-Reviewed Content in 2020: COVID-19 and Chronic Disease, Public Health and Pharmacy, Eliminating Health Disparities, Global Health, and Student Research

**DOI:** 10.5888/pcd17.200447

**Published:** 2020-09-24

**Authors:** Leonard Jack

**Affiliations:** 1 Preventing Chronic Disease, Office of Medicine and Science, National Center for Chronic Disease Prevention and Health Promotion, Centers for Disease Control and Prevention, Atlanta, Georgia

This year has been challenging in numerous ways, and it has been imperative for all of us in the public health field to respond decisively to the COVID-19 pandemic emergency in the short-term and in the long-term to look for new ways to address health disparities that have been highlighted by COVID-19. Now more than ever, *Preventing Chronic Disease* (*PCD*) is committed to its mission to provide peer-reviewed content that promotes dialogue among researchers, practitioners, and policy makers worldwide and advances the field of public health as a whole. *PCD* has seen the success of its efforts reflected in the annual increase in the journal’s impact factor. In July 2020, *PCD* received its new Journal Impact Factor of 2.144, a jump from 2.038 the previous year in the Web of Science Journal Citation Reports. *PCD’*s other metrics are also strong: the journal has a Scimago Journal and Country Rank of 22 out of 145 US journals in the category of Public, Environmental and Occupational Health. *PCD* is also ranked third of 19 open access US journals in this category.

We are grateful to the authors, peer reviewers, and readers who have contributed to the journal’s continuing success. In turn, *PCD* has sought even more opportunities in 2020 to publish timely content that contributes to the field at a critical time. This year *PCD* is publishing peer-reviewed content in 3 collections around timely topics that address 1) the public health response to COVID-19 and chronic disease, 2) the role of pharmacy and public health in improving population health, and 3) insights into research and evaluation that improve oral health among people who are at increased or higher risk for chronic conditions.

The COVID-19 pandemic presents a worldwide public health challenge to understand not only how to mediate its spread but also how to continue maintaining and promoting chronic disease management and control. The impact of COVID-19 on the overall health of individuals who are at increased risk for or living with chronic conditions such as cardiovascular disease, diabetes, and chronic lung disease has become even more apparent, because these conditions contribute to poorer health outcomes and mortality (1,2). *PCD* recognizes that a great amount can be learned from efforts occurring around the world; one discipline alone cannot offer solutions to this complex global public health threat. The journal brought together a wide variety of perspectives and expertise from around the world to share emerging public health approaches that address the intersection of COVID-19 and chronic disease.

In early August 2020, *PCD* published a special supplement entitled “US Public Health Response to COVID-19 and Chronic Disease: Continuing the Commitment to Improve Population Health,” (3) featuring 16 peer-reviewed commentaries addressing a wide range of topics: the persistent toll exacted by social determinants of health on health outcomes, understanding sex difference in COVID-19–related mortality among men, population health approaches to behavioral health during and after COVID-19, the role of culture in communicating risk, reaching Hispanic and African-American communities through existing chronic disease infrastructures, the rural health response to COVID-19, contributions of community pharmacists to disease management during COVID-19, ensuring access to oral health during COVID-19, and preparing students with classroom and practice experience to participate in ongoing public health responses to COVID-19. Content appearing in this supplement can be accessed at https://www.cdc.gov/pcd/collections/PCD_Special_Supplement_2020.htm.

In addition to this recently released supplement, *PCD* plans to publish peer-reviewed content generated by experts in medicine, public health, psychology, health systems, community engagement, school health, infectious disease, nursing, pharmacy, oral health, environmental health, and other areas related to COVID-19 and chronic disease. *PCD* is interested in continuing to receive submissions from around the world that provide timely insights around the topics featured in the supplement. Interested authors can use any of the journal’s article types (https://www.cdc.gov/pcd/for_authors/types_of_articles.htm). In advance of submitting a manuscript, authors are invited to submit a brief inquiry to determine whether the proposed topic would be within the scope and interest of the journal. For more information on how and where to submit an inquiry, please visit the *PCD* author web page (https://www.cdc.gov/pcd/for_authors/submit_inquiry.htm).

Preventing and controlling chronic disease remains a global public health challenge. Over the past decades, a range of innovative community- and clinical-driven strategies in public health and pharmacy have evolved to prevent and reduce the burden of chronic conditions. Today, *PCD* is pleased to release a new collection around this important topic, “Public Health and Pharmacy: Collaborative Approaches to Improve Population Health.” This collection features 15 peer-reviewed articles providing timely information on effective ways these disciplines are working collaboratively to improve the nation’s health. The articles in this collection provide examples of pharmacies and pharmacists improving population health through health screenings and disease management (diabetes, cholesterol, blood pressure) in health care and community settings. Content appearing in this supplement can be accessed at https://wwwdev.cdc.gov/pcd/collections/Public_Health_and_Pharmacy.htm. *PCD* also welcomes future submissions addressing similar topics featured in this collection; for more information on how and where to submit an inquiry, please visit https://www.cdc.gov/pcd/for_authors/submit_inquiry.htm.

*PCD* is also seeking to publish more peer-reviewed articles that identify ways to improve access to care, eliminate health disparities, and understand the importance of public health infrastructure as it relates to oral health. To that end, *PCD* is planning to release a collection of articles focusing on tooth loss in a Medicaid adult population, predictors of oral health behaviors among low-income children, changes in health disparities in use of dental care among US children and adolescents, impact of increased reimbursement for preventive dental care on tertiary oral health, and more. This collection will feature several peer-reviewed articles and is scheduled for release in December 2020.

In addition to these 2 important collections, *PCD* would like to bring 3 future collections to your attention as an author, reader, or both. *PCD* recently launched a Call for Papers on a collection entitled “Global Perspectives on Improving Health and Well-Being in Diverse Settings.” Submissions for this collection are due no later September 30, 2020. *PCD* has received submissions from around the world and is interested in learning about a range of community-based, clinically driven, and technology-informed innovations for strategies used to prevent and reduce the burden of chronic conditions worldwide. Papers accepted for this collection will be published on a rolling basis, and all accepted papers will be bundled into a collection in 2021.

*PCD* has announced another Call for Papers for an upcoming collection that focuses on the impact of chronic disease on poor health outcomes, reduction in quality of life, and increases in health care costs. This collection, entitled “Addressing Health Disparities and Improving Population Health in Diverse Communities and Settings,” seeks submissions addressing a range of topics that include but are not limited to engaging diverse communities in identifying and implementing shared community solutions that address local health concerns; leveraging health care systems, including hospitals, health care centers, and health plans, and the community to facilitate greater improvements in population health; communicating health risk reduction practices to various audiences (eg, general population, low-literacy, English as a second language); developing the infrastructure necessary to establish appropriate methods to collect, track, and report health outcomes; and other related areas. Authors interested in submitting manuscripts to the journal for consideration may do so on or before the December 31, 2020, due date. Accepted papers are being published on a rolling basis, with the final collection published in 2021.

*PCD’s* third Call for Papers centers on students at the high school, undergraduate, and graduate levels, and recent post-graduates, and invites students to submit papers relevant to the prevention, screening, surveillance, and population-based interventions in chronic diseases, including but not limited to arthritis, asthma, cancer, psychological health, diabetes, obesity, and cardiovascular disease. Accepted manuscripts will appear in the *PCD* Student Research Collection. The primary goal of the collection is to provide an opportunity for students to become familiar with a journal’s submissions and peer-review process. Over the years, *PCD* has offered students opportunities to further develop their science-writing abilities by submitting research papers to the journal for consideration. *PCD* has received hundreds of papers, all of which undergo the journal’s standard peer-review process. Submissions for this year’s Call for Student Research Papers are due by Friday, December 11, 2020. Students interested in submitting research-oriented papers must be sure to read and follow all submission requirements. Details on the student publishing opportunity can be found on our website at https://www.cdc.gov/pcd/announcements.htm.

Please stay tuned as the journal publishes content of interest to its readers and identifies future timely topics around which authors can submit manuscripts for consideration. Stay up to date on the latest calls for papers and *PCD* collections. Receive the latest articles and journal news directly in your inbox by subscribing to the journal through CDC.gov. We thank you for your continued support of the journal. Your support ensures that *PCD* can continue to remain a viable resource to researchers, evaluators, and policy makers worldwide.
